# Risk factors for placental malaria, sulfadoxine-pyrimethamine doses, and birth outcomes in a rural to urban prospective cohort study on the Bandiagara Escarpment and Bamako, Mali

**DOI:** 10.1186/s12936-022-04125-6

**Published:** 2022-03-31

**Authors:** Claudius Vincenz, Zachary Dolo, Serou Saye, Jennie L. Lovett, Beverly I. Strassmann

**Affiliations:** 1 Research Center for Group Dynamics, Institute for Social Research, University of Michigan, Ann Arbor, MI USA; 2 Independent Investigator, Bandiagara Cercle, Mali; 3 Department of Anthropology, University of Michigan, Ann Arbor, MI USA

**Keywords:** Placenta, Malaria, Pregnancy, Histology, Birth weight, IPTp, Sulfadoxine-pyrimethamine, Maternal education, Birth seasonality, Cohort

## Abstract

**Background:**

Malaria in Mali remains a primary cause of morbidity and mortality, with women at high risk during pregnancy for placental malaria (PM). Risk for PM and its association with birth outcomes was evaluated in a rural to urban longitudinal cohort on the Bandiagara Escarpment and the District of Bamako.

**Methods:**

Placental samples (N = 317) were collected from 249 mothers who were participants in a prospective cohort study directed by BIS in the years 2011 to 2019. A placental pathologist and research assistant evaluated the samples by histology in blinded fashion to assess PM infection stage and parasite density. Generalized estimating equations (GEE) were used to model the odds of PM infection.

**Results:**

In a multivariable model, pregnancies in Bamako, beyond secondary education, births in the rainy season (instead of the hot dry season), and births to women who had ≥ 3 doses of sulfadoxine-pyrimethamine (SP) instead of no doses were associated with reduced odds of experiencing PM (active and past infections combined). Births in later years of the study were strongly associated with reduced odds of PM. Maternal age, which was positively associated with offspring year of birth, was significant as a predictor of PM only if offspring year of birth was omitted from the model. Gravidity was positively associated with both maternal age and offspring year of birth such that if either variable was included in the model, then gravidity was no longer significant. However, if maternal age or year of offspring birth were not adjusted for, then the odds of PM were nearly two-fold higher in primigravida compared to multigravida. Birth outcomes improved (+ 285 g birth weight, + 2 cm birth length, + 75 g placental weight) for women who had ≥ 3 doses of SP compared to no doses, but no difference was detected in birth weight or length for women who had 2 instead of ≥ 3 SP doses. However, at 2 instead of ≥ 3 doses placentas were 36 g lighter and the odds of low birth weight (< 2500 g) were 14% higher. Severe parasite densities (> 10% erythrocytes infected) were significantly associated with decreases in birth weight, birth length, and placental weight, as were chronic PM infections. The women who received no SP during pregnancy (7% of the study total) were younger and lacked primary school education. The women who received ≥ 3 doses of SP came from more affluent families.

**Conclusions:**

Women who received no doses of SP during pregnancy experienced the most disadvantageous birth outcomes in both Bamako and on the Bandiagara Escarpment. Such women tended to be younger and to have had no primary school education. Targeting such women for antenatal care, which is the setting in which SP is most commonly administered in Mali, will have a more positive impact on public health than focusing on the increment from two to three doses of SP, although that increment is also desirable.

**Supplementary Information:**

The online version contains supplementary material available at 10.1186/s12936-022-04125-6.

## Background

In 33 countries in sub-Saharan Africa in 2019, more than eleven million pregnant women were exposed to malaria infections and delivered 882,000 low birthweight neonates [1]. During pregnancy, erythrocytes infected with mature asexual *Plasmodium falciparum* parasites sequester in the placenta. Placental malaria (PM) is defined by the presence of infected erythrocytes (IE) or haemozoin in the placental intervillous space and is associated with maternal illness and anaemia [2–4], low birth weight [3, 5], and preterm birth [3, 5].

To prevent malaria infections in pregnant women, the World Health Organization (WHO) recommends a combination of intermittent preventive treatment in pregnancy (IPTp) with sulfadoxine-pyrimethamine (SP) as part of antenatal care (ANC), long-lasting insecticidal bed nets (LLINs) [3], and indoor residual spraying (IRS) [6]. Doses of SP are to be administered one month apart at the start of the second trimester [6] and have been effective in reducing the risk of placental malaria, low birth weight, and severe maternal anaemia [7]. Eleven sub-Saharan African countries account for about 70% of the global malaria case burden and estimated deaths [3]. One of these countries is Mali, which has among the highest malaria case incidence rates at > 250 per 1000 population at risk [3]. In 2013, the Malian Ministry of Health revised its guidelines to reflect the WHO’s recommendation that at least three doses of SP should be administered to pregnant women beginning in the second trimester [8]. Despite national control efforts, in 2018 only 42% of women received the recommended three doses of SP [9], leaving a significant number of pregnant women at risk for malaria infections.

The women in this prospective cohort study were enrolled in infancy or early childhood (median age 1.36 years) on the Bandiagara Escarpment in central Mali. This 21-year study is unique for Africa in that follow-up occurred on a regular, nearly annual basis to adulthood. It is rare for an observational study that compares urban and rural participants to have a quasi-experimental design. In this study, follow-up occurred both in the rural community and after migration to Bamako, permitting comparison of risk factors in the same cohort and same ethnicity (the Dogon) in both locations. This study is the first to assess risk factors for PM in Mali using placental tissue histology, and the results are helpful for tracking the success of the National Malaria Control Program (NMCP) on the Bandiagara Escarpment, Mali. This study addressed the following questions: (1) Was risk for PM higher in Bamako or on the Bandiagara Escarpment? (2) What were the maternal risk factors for PM in this cohort? (3) What was the association between number of IPTp-SP doses, PM, and birth outcomes—in particular, were there observable benefits for three instead of two doses of SP? (4) What factors predicted how many IPTp-SP doses women received?

## Methods

### Study population and design

**Fig. 1:**
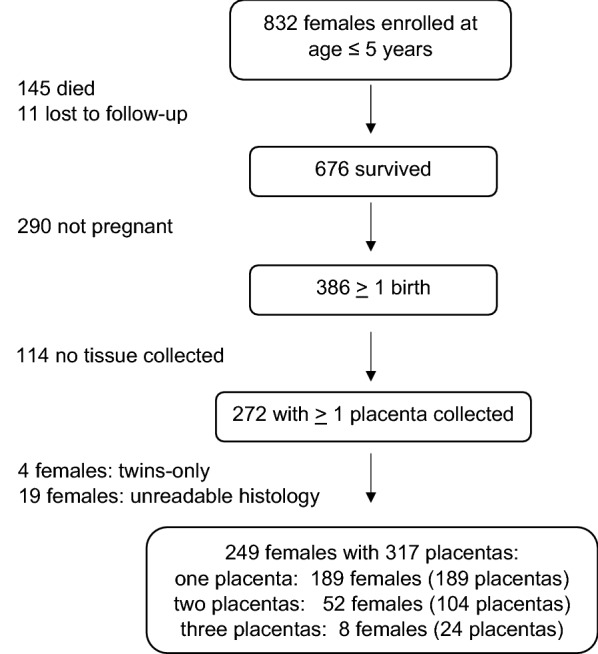
Profile of females in the cohort study who were enrolled at age ≤ 5 years and who survived to maturity and who contributed placentas for histological assessment

Study participants (N = 832 females) belong to the Dogon ethnic group and were enrolled by BIS in a prospective cohort study during infancy or early childhood in nine rural villages on the plateau of the Bandiagara Escarpment [10, 11] in the Region of Mopti. Enrollment took place from 1998 to 2000 at a mean age of 1.36 years (range 0 to 5 years). Follow-up occurred in 1999, 2000, 2004, 2007, and annually from 2010 through 2019. From 2010 onward, annual follow-up, including interviews and measurements, occurred in both the villages and in Bamako, with retention of all but 6% of the migrants to that city. The profile of the study is shown in Fig. 1. A total of 145 girls died (mostly in infancy and very early childhood), 11 were lost to follow-up, and 676 survived to adolescence and adulthood and remained in the study (retention rate of 81% over 21 years). Among the survivors, 386 gave birth, with placental tissue collected and analyzed from 249 mothers who provided a total of 317 placentas from singleton births (Fig. 1) in the years 2011 to 2019.

### Study setting

#### Placental collection

In Bamako, placentas were collected at the Centre Hospitalier Universitaire Gabriel Touré, the Centre de Santé de Référence Commune V and VI, and other health facilities. In the villages, placentas were collected at a rural hospital that serves over 50 villages along the Bandiagara Escarpment. At this rural hospital, the midwives were trained to identify study participants who had consented to placental collections prior to labour. In Bamako, a research physician from the same ethnicity traveled to the hospital upon being informed by a woman or her family that she had initiated labour. At all locations, staff who performed placental collections were different from the medical personnel who facilitated deliveries. Services offered to the women in Bamako included free antenatal visits, detailed bloodwork at the Institut Merieux, and ultrasounds, whereas in the villages only antenatal visits were feasible. In both locations, uptake was uneven. As Bamako is a large city, transportation was provided in a private vehicle or taxi.

#### Malaria prevalence/incidence

*Plasmodium falciparum* prevalence in children age 6–59 months during the study period in the Mopti Region was 65.8% in 2012–2013 and declined to 24.9% in 2018; In Bamako, the corresponding prevalence was 6.2% in 2012–2013 and declined to 2.9% in 2018 [9]. Similarly, the estimated all-age incidence of *P. falciparum* per 1000 people per annum declined from 575 in 2014 to 176 in 2018 in the Mopti Region, and in Bamako declined from 193 in 2014 to 79 in 2018 [12].

### Histology

Biological specimens were collected from placentas within 30 min of placental expulsion. Specimens for histology and nucleic acid analysis were obtained simultaneously from the two faces of a well-formed cotyledon using a mirrored sampling approach. The tissue sampled (< 1cm^3^) was from the central two-thirds of the placental surface and consisted mainly of fetal villi and maternal intervillous space. Two histological samples were dissected from two cotyledons for each placenta and fixed with 37% formalin freshly diluted 1:10 with buffered saline. Fixation was for 36 h on ice followed by a 30 min wash in 70% ethanol followed by storage in a – 20 °C solar freezer for up to 18 months. The samples were shipped to the University of Michigan on dry ice. Upon arrival, tissues were stored at −80 °C, then thawed in 70% ethanol for mounting, sectioning, and haematoxylin and eosin (H&E) staining at the University of Michigan Tissue and Molecular Core (TMP). Giemsa staining was performed in the Strassmann laboratory. Blinded histological evaluation was performed by a placental pathologist and research assistant using established guidelines for assessment of placental malaria [13, 14]. Discrepancies between evaluators were resolved by a consensus evaluation by both parties. Slides were scored for 6 characteristics: malaria parasites (presence and density), haemozoin pigment (presence and density), and malaria infection (none, acute, chronic, past).

### Definitions

Placental malaria infection stages were classified based on histopathology as: uninfected (no parasites or pigment present); acute (parasites present in maternal erythrocytes in the intervillous space, pigment in erythrocytes and monocytes in the intervillous space but no pigment in fibrin or cells within fibrin); chronic (parasites in maternal erythrocytes in the intervillous space and pigment in erythrocytes and circulating monocytes within the intervillous space and pigment in fibrin or cells within fibrin and/or chorionic villous syncytiotrophoblast or stroma) and past (pigment confined to fibrin or cells within fibrin but no parasites present) [14]. Active infections included both acute and chronic infections but not past infections. Density of infected erythrocytes (parasitaemia) was classified as not present, mild (< 1% maternal erythrocytes infected), moderate (1–10% of maternal erythrocytes infected), or severe (> 10% maternal erythrocytes infected) in 20–40 fields at 40-100X magnification. Gravidity was categorized as primigravid (women’s first pregnancy) or multigravid (women’s second or later pregnancies). Low birth weight (LBW) infants weighed < 2500 g. As data on gestational age at delivery were not available, for the purpose of estimating the trimester in which SP doses were administered, the week of pregnancy was calculated by subtracting 40 weeks from the date of delivery.

### Clinical data

Prenatal care was available to women in both Bamako and the rural villages, although participation was uneven and often did not comply with WHO guidelines [13]. During antenatal care (ANC), Malian clinicians measured the mothers’ height, weight, and blood pressure, estimated due dates, administered elemental iron, and low dose folic acid. They also administered SP, and SP dosing was obtained from patient medical records as opposed to using self-reports. A total of 232 of the 317 pregnancies in the analysis (73.2%) included testing for HIV during prenatal exams and there was one positive result. Births were by vaginal delivery followed by administration of oxytocin to assist expulsion of the placenta. Birth parameters were measured by midwives immediately after birth and included newborn length, birth weight, and placental weight. No births to women in this study took place outside of healthcare facilities.

### Covariates

Field data were collected by BIS and CV and a trained team of Malian collaborators (ZD, SS). Body mass index (BMI), educational attainment, and location of residence for each participant were obtained during the approximately annual follow-up of cohort members. Wealth z-scores were determined using a system of independent rankings by approximately five judges from each study village who ranked each family’s wealth relative to that of other families in the same village. Residence was recorded as rural if the woman lived in one of the original villages or urban if she had moved to Bamako. Women who moved during gestation were classified in the location where they lived during the majority of the pregnancy. During annual interviews, no participants reported being smokers (current or past), in agreement with cultural norms for Dogon women and observations by the field team and authors (ZD, SS, BIS, CV) who interacted regularly with these women.

### Statistical analyses

IBM SPSS v. 27 was used to generate risk factor models for the odds of PM (yes/no) or low birth weight (< 2500 or ≥ 2500 g) using general estimating equations (GEE) for binary logistic regression that took into account the non-independence of maternal siblings as some women contributed more than one placenta/offspring to the study. Logistic GEE models were also used to assess the characteristics of women who were fully compliant with the policy that women should receive at least 3 doses of SP, as well to gain insight into the women who received no doses of SP. Lastly, GEE was used to model birth outcomes as continuous dependent variables (birth weight in g, birth length in cm, or placenta weight in g). *P* values < 0.05 were considered statistically significant. All models were adjusted for pertinent covariates as described below.

## Results

### Study participant characteristics

**Fig. 2:**
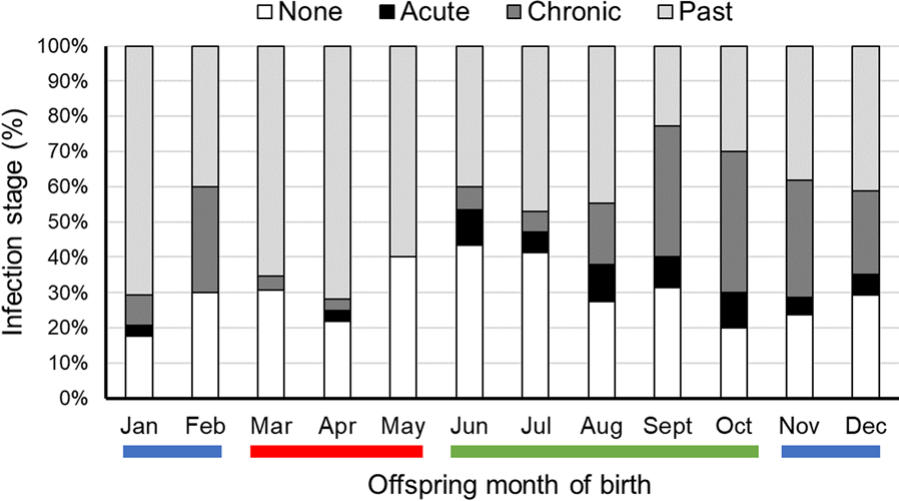
PM infection stages by offspring month of birth for all study years. Seasons are indicated by colored bars as cool/dry (blue), hot/dry (red), and rainy (green)

Descriptive statistics for the mothers, births, and for placental malaria are shown in Table 1. About half of the placentas were from first births (56% of mothers were primigravida and 44% were multigravida) and the majority of mothers lived in rural villages on the Bandiagara Escarpment (84% in villages, 16% in Bamako) during most of gestation (Table 1). Mean (SD) maternal age at delivery was 20.4 ± 2.2 years and mean pre-pregnancy BMI was 21.8 ± 2.4. Twenty-six percent of the births in the study were to mothers who reported no schooling, 38% reported some primary education, 25% reported some secondary education, and 11% reported some education beyond secondary school. Seven percent of the women received no doses of SP, 32% received one dose, 36% received two doses, and 25% received three or more doses of SP. The distribution of births by season was 45% in the rainy season, 30% in the cool dry season, and 25% in the hot dry season. PM stages for each month of the study are shown in Fig. 2 and Additional file 3: Fig. S3. The mean (SD) birth weight (g) was 2719 (405), birth length (cm) was 49.1 (1.7), and placenta weight (g) was 483 (91). Twenty-five percent of the neonates were of low birth weight (< 2500 g) and 75% were of normal birth weight (Table 1).

**Table 1:** Maternal, pregnancy, and birth characteristics (317 mother–offspring pairs)

Categorical variables	Village *n* (%)	Bamako *n* (%)	Total *n* (%)
Mothers			
Residence during pregnancy	268 (84.5)	49 (15.5)	317 (100)
Education			
No Schooling	71 (26.5)	12 (24.5)	83 (26.2)
Some primary	108 (40.3)	12 (24.5)	120 (37.9)
Some secondary	64 (23.9)	16 (32.7)	80 (25.2)
Beyond secondary	25 (9.3)	9 (18.4)	34 (10.7)
IPTp-SP doses			
None	19 (7.1)	2 (4.1)	21 (6.6)
1	84 (31.3)	19 (38.8)	103 (32.5)
2	101 (37.7)	14 (28.6)	115 (36.3)
3 +	64 (23.9)	14 (28.6)	78 (24.6)
Parity			
Primigravida	150 (56.0)	26 (53.1)	176 (55.5)
Multigravida	118 (44.0)	23 (46.0)	141 (44.5)
Births			
Survival status			
Survived	263 (98.1)	47 (95.9)	310 (97.8)
Stillborn	4 (1.5)	2 (4.1)	6 (1.9)
Missing	1 (0.4)	0	1 (0.3)
Sex			
Female	131 (48.9)	19 (38.8)	150 (47.3)
Male	137 (51.1)	30 (61.2)	167 (52.7)
Weight^a^			
Low (< 2500 g)	77 (29.3)	1 (2.1)	78 (25.2)
Normal (≥ 2500 g)	186 (70.7)	46 (97.9)	232 (74.8)
Season			
Hot dry (Mar–May)	71 (26.5)	8 (16.3)	79 (24.9)
Rainy (Jun–Oct)	117 (43.7)	27 (55.1)	144 (45.4)
Cool dry (Nov–Feb)	80 (29.9)	14 (28.6)	94 (29.7)
Year			
2011	2 (0.7)	0	2 (0.6)
2012	1 (0.4)	0	1 (0.3)
2013	1 (0.4)	0	1 (0.3)
2014	37 (13.8)	1 (2.0)	38 (12.0)
2015	41 (15.3)	1 (2.0)	42 (13.2)
2016	54 (20.1)	10 (20.4)	64 (20.2)
2017	51 (19.0)	20 (40.8)	71 (22.4)
2018	57 (21.3)	11 (22.4)	68 (21.5)
2019	24 (9.0)	6 (12.2)	30 (9.5)
Placental malaria			
Infection stage			
None	63 (23.5)	27 (55.1)	90 (28.4)
Acute	17 (6.3)	0	17 (5.4)
Chronic	53 (19.8)	2 (4.1)	55 (17.4)
Past	129 (48.1)	20 (40.8)	149 (47.0)
Missing	6 (2.2)	0	6 (1.9)
Parasite density			
None	194 (72.4)	47 (95.9)	241 (76.0)
Mild	32 (11.9)	1 (2.0)	33 (10.4)
Moderate	17 (6.3)	0	17 (5.4)
Severe	20 (7.5)	1 (2.0)	21 (6.6)
Missing	5 (1.9)	0	5 (1.6)

Quantitative variables	Village mean (SD)	Bamako mean (SD)	Total mean (SD)
Maternal age at delivery (years)	20.3 (2.2)	21.2 (1.9)	20.4 (2.2)
Maternal pre-pregnancy BMI	21.6 (2.3)	22.9 (2.7)	21.8 (2.4)
Birth weight (g)^a^	2680.1 (402.8)	2937.4 (344.2)	2719.1 (404.6)
Birth length (cm)^a^	49.1 (1.8)	49.4 (1.3)	49.1 (1.7)
Placenta weight (g)^a^	478.4 (92.7)	510.0 (75.5)	483.2 (90.9)

^*a*^for 310 livebirths. *BMI* body mass index, *SD* standard deviation

### PM prevalence in study specimens

Of 317 placentas analyzed, 90 (28%) had no indicators of active or past PM, and 6 (2%) could not be scored (Table 1). Among the 72 (23%) active PM infections, 17 were scored as acute (parasites but no haemozoin visible) and 55 were scored as chronic (haemozoin and parasites visible). There were 149 (47%) past PM infections indicated by the presence of haemozoin but not parasites. Further, among the 317 placentas, 76% showed no evidence of malaria parasites, and 2% could not be scored. Parasitaemia in the remaining samples was mild 10%, moderate 5%, and severe 7% (Table 1).

### Maternal risk factors for PM (active & past Infections combined)

Models of the risk factors for active and/or past PM infections are shown in Table 2. Models 1 and 2 differ in that model 2 omits year of offspring birth, which is correlated with maternal age (Additional file 1: Fig. S1). Maternal age was not associated with PM in Model 1. In Model 2, which excluded year of offspring birth, a one-year increase in maternal age was associated with a 23% decrease (p = 0.007) in the odds of PM. A one unit increase in maternal pre-pregnancy BMI was associated with 13% lower odds (p = 0.048) of PM infection in Model 1 and 9% lower odds in Model 2, but the latter finding did not meet the threshold for statistical significance (p = 0.176).

**Table 2:** Multivariable models of the maternal risk factors for PM infection (active and past infections combined) (N = 313)

	Model 1				Model 2			
	OR	95% CI		p-value	OR	95% CI		p-value
		Lower	Upper			Lower	Upper	
Maternal age (years)	0.99	0.79	1.23	0.908	0.77	0.64	0.93	**0.007**
Pre-pregnancy BMI	0.87	0.76	1.00	**0.048**	0.91	0.79	1.04	0.176
Gravidity (ref. Multigravid)								
Primigravid	1.44	0.73	2.85	0.293	1.11	0.60	2.06	0.743
IPTp-SP Doses (ref. 3 +)								
None	14.57	3.20	66.43	** < 0.001**	8.20	2.09	32.3	**0.003**
1	2.06	0.96	4.43	0.064	2.29	1.14	4.57	**0.019**
2	1.55	0.72	3.31	0.263	1.63	0.81	3.30	0.171
Residence during pregnancy (ref. Village)								
Bamako	0.33	0.14	0.81	**0.015**	0.33	0.15	0.71	**0.005**
Wealth z-score	1.29	0.91	1.82	0.157	1.37	0.99	1.91	0.060
Education (ref. Never attended)								
Primary	0.53	0.24	1.19	0.126	0.63	0.30	1.32	0.221
Secondary	1.33	0.54	3.31	0.537	1.16	0.51	2.65	0.727
Beyond secondary	0.17	0.06	0.46	**0.001**	0.25	0.10	0.65	**0.004**
Season of birth (ref. Hot Dry, Mar–May)								
Rainy, Jun–Oct	0.23	0.09	0.58	**0.002**	0.69	0.34	1.39	0.302
Cool Dry, Nov–Feb	0.45	0.18	1.10	0.081	1.02	0.46	2.25	0.961
Year of offspring birth (ref. 2018)								
2014	4.19	1.05	16.72	**0.043**				
2015	8.16	2.32	28.67	**0.001**				
2016	7.04	2.54	19.49	** < 0.001**				
2017	2.50	1.00	6.25	0.051				
2019	0.26	0.09	0.82	**0.021**				

Odds Ratios (OR) and 95% confidence intervals (CI) from general estimating equations for logistic regression, p < 0.05 bolded. Model 2 excludes year of birth. *ref* reference group, *IPTp-SP* intermittent preventative treatment in pregnancy with sulfadoxine-pyrimethamine. Intercepts were included (not shown)

Primigravid women had a higher risk of PM than did multigravid women in both Models 1 and 2, but the difference was not statistically significant. Gravidity was correlated with both maternal age and offspring year of birth (Additional file 2: Fig. S2) such that if either variable was included in the model, gravidity was no longer significant. However, if the model did not adjust for maternal age or year of offspring birth, then the odds of PM (active and past combined) were 1.88 fold higher (p = 0.023) in primigravida compared to multigravida (Table S1). Pre-pregnancy BMI was not strongly associated with gravidity (Additional file 2: Fig. S2). Mean (SD) BMI in primigravida was 21.5 (2.4) and in the multigravida was 22.2 (2.5). In no model for active infections was gravidity significant (p > 0.5).

Risk of PM in both models 1 and 2 (Table 2) tended to increase as doses of SP received decreased. Compared to 3 or more doses of SP, getting no doses of SP was associated with 14-fold and eight-fold higher odds of PM in models 1 and 2, respectively (Model 1: OR = 14.57, p = 0.001; Model 2: OR = 8.20, p = 0.003). Receiving one dose instead of three doses was associated with an approximately two-fold higher odds of PM in both models (Model 1: OR = 2.06, p = 0.064; Model 2: OR = 2.29, p = 0.019), but this association was statistically significant only in Model 2. Women receiving two doses of SP compared to those receiving three doses had an approximately 50% increased odds of infection but this increase was not statistically significant in either model (Model 1: OR = 1.55, p = 0.263; Model 2: OR = 1.63, p = 0.171).

Residence in Bamako instead of the villages during pregnancy was associated with 67% lower odds of PM in both models, (Model 1: OR 0.33, p = 0.015, Model 2: OR 0.33, p = 0.005). Family wealth z-score was not significantly associated with the odds of placental malaria (Model 1: OR = 1.29, p = 0.16, Model 2: OR = 0.32, p = 0.06). Compared with women who had no schooling, women who went beyond secondary school had 83% and 75% decreased odds of PM in Models 1 and 2, respectively (Model 1: OR = 0.17, p = 0.001, Model 2: OR = 0.25, p = 0.004). Births during the rainy season compared to the hot dry season were associated with 77% decreased odds of PM in Model 1 (p = 0.002) and 31% decreased odds in model 2 that was not statistically significant (p = 0.30). The odds of PM for births occurring during cool dry months were not significantly different from the odds of PM for the hot dry months (Model 1: OR = 0.45, p = 0.081, Model 2: OR = 1.02, p = 0.961). Compared with deliveries in 2018, births in 2014, 2015, 2016, and 2017 had two to eight-fold higher odds of PM and deliveries in 2019 had 74% lower odds of PM.

### Maternal risk factors for active PM

The risk factors for active PM are shown in Table 3. Model 4 differs from Model 3 in that it does not include year of offspring birth, which is correlated with maternal age (Additional file 1: Fig. S1), as discussed above. In Model 3, maternal age was not associated with active PM (OR = 0.96, p = 0.696). In Model 4, a one-year increase in maternal age was associated with a 17% decrease (OR = 0.83, p = 0.016) in the odds of active PM. Pre-pregnancy BMI, gravidity, wealth z-score, and education were not significantly associated with active PM in models 3 or 4. As was the case for active and past infections combined, the odds of active PM tended to increase as doses of SP received decreased. Compared with women who received three doses of SP, women who had no doses had ten-fold and six-fold higher odds of active PM in models 3 and 4, respectively (Model 3: OR = 10.50, p = 0.001; Model 4: OR = 5.90, p = 0.008) and women who had only one dose had more than two-fold higher odds of active PM (Model 3: OR = 2.64, p = 0.036, Model 4: OR = 2.57, p = 0.030). The difference between three doses and two doses was not statistically significant (Model 3: OR = 1.49, p = 0.411; Model 4: OR = 1.35, p = 0.513).

**Table 3:** Multivariable models of maternal risk factors for active PM infection (N = 313)

	Model 3				Model 4			
	OR	95% CI		p-value	OR	95% CI		p-value
		Lower	Upper			Lower	Upper	
Maternal age (years)	0.96	0.79	1.17	0.696	0.83	0.71	0.96	**0.016**
Pre-pregnancy BMI	0.97	0.84	1.11	0.640	0.97	0.83	1.12	0.670
Gravidity (ref. Multigravid)								
Primigravid	0.79	0.37	1.72	0.556	0.80	0.38	1.66	0.547
IPTp-SP doses (ref. 3−)								
None	10.50	2.65	41.63	**0.001**	5.90	1.60	21.78	**0.008**
1	2.64	1.07	6.55	**0.036**	2.57	1.09	6.03	**0.030**
2	1.49	0.57	3.88	0.411	1.35	0.55	3.29	0.513
Residence during pregnancy (ref. Village)								
Bamako	0.13	0.03	0.73	**0.020**	0.11	0.02	0.52	**0.005**
Wealth z-score	1.25	0.87	1.80	0.230	1.26	0.90	1.76	0.184
Education (ref. Never attended)								
Primary	0.60	0.27	1.32	0.206	0.63	0.30	1.32	0.224
Secondary	0.80	0.32	2.00	0.637	0.71	0.32	1.61	0.417
Beyond secondary	0.59	0.18	1.88	0.371	0.68	0.22	2.13	0.511
Season of birth (ref. Hot Dry, Mar–May)								
Rainy, Jun–Oct	11.00	3.27	37.01	** < 0.001**	14.33	4.34	47.27	** < 0.001**
Cool Dry, Nov–Feb	7.02	2.08	23.70	**0.002**	7.95	2.28	27.78	**0.001**
Year of offspring birth (ref. 2018)								
2014	4.98	1.47	16.88	**0.010**				
2015	7.04	2.20	22.49	**0.001**				
2016	4.90	1.62	14.86	**0.005**				
2017	1.23	0.34	4.42	0.754				
2019	0.82	0.08	8.61	0.871				

Odds Ratios (OR) and 95% confidence intervals (CI) from general estimating equations for logistic regression, p < 0.05 bolded. Model 4 excludes year of birth. *ref* reference group, *CI* confidence interval, *IPTp-SP* intermittent preventative treatment in pregnancy with sulfadoxine-pyrimethamine. Intercepts were included (not shown)

For women who lived in Bamako instead of the villages during their pregnancies, the odds of active infections were 87% lower in Model 3 and 89% lower in model 4 (Model 3: OR = 0.13, p = 0.020, Model 4: OR = 0.11, p = 0.005). Births during the rainy season were associated with eleven and fourteen times higher odds ratios for active PM compared to births in the hot dry season in models 3 and 4, respectively (Model 3: OR = 11.00, p < 0.001, Model 4 OR = 14.33, p < 0.001). The odds of active PM were about five to seven-fold higher for births in 2014, 2015, and 2016 relative to 2018.

### Associations between infection stage, parasite density, SP doses, and birth outcomes

#### Infection stage

Models relating PM infection stage to birth outcomes (birth weight, birth length, and placenta weight) are shown in Table 4. All models adjust for maternal age and pre-pregnancy BMI, gravidity, sex of offspring, and residence (Bamako or village). If the placenta showed histological evidence of chronic infection (N = 55) as opposed to no infection (N = 90), birth weight was 313 g lower (p < 0.001), birth length was 0.66 cm shorter (p = 0.035), and placental weight was 68 g lower (p < 0.001). Acute (N = 17) and past infections (N = 149) were not significantly associated with any of the three birth parameters.

**Table 4:** Associations between PM infection stage, parasite density, IPTp-SP doses, and birth outcomes in nine multivariable models

	Birth weight (g)				Birth Length (cm)				Placenta weight (g)			
		95% CI				95% CI				95% CI		
	B	Lower	Upper	p-value	B	Lower	Upper	p-value	B	Lower	Upper	p-value
Infection stage (ref. None)												
N = 305					N = 305				N = 302			
Acute	32.85	− 139.64	205.34	0.709	0.06	− 0.58	0.70	0.864	15.24	− 30.91	61.39	0.518
Chronic	− 313.05	− 437.31	− 188.80	** < 0.001**	− 0.66	− 1.26	− 0.05	**0.035**	− 67.68	− 97.54	− 37.82	** < 0.001**
Past	− 31.58	− 120.69	57.53	0.487	0.07	− 0.37	0.51	0.755	− 21.11	− 44.90	2.68	0.082
Parasite density (ref. None)												
N = 305					N = 305				N = 302			
Mild	− 80.40	− 229.30	68.50	0.290	− 0.28	− 0.90	0.34	0.380	− 20.21	− 51.52	11.10	0.206
Moderate	− 228.50	− 414.32	− 42.68	**0.016**	− 0.09	− 0.88	0.71	0.830	− 39.58	− 84.26	5.10	0.082
Severe	− 410.35	− 548.07	− 272.64	** < 0.001**	− 1.37	− 2.12	− 0.63	** < 0.001**	− 64.96	− 101.39	− 28.53	** < 0.001**
IPTp-SP doses (ref. 3 +)												
N = 310					N = 310				N = 307			
none	− 284.72	− 545.65	− 23.79	**0.032**	− 2.04	− 3.43	− 0.65	**0.004**	− 75.39	− 124.16	− 26.62	**0.002**
1	− 75.49	− 185.82	34.83	0.180	− 0.49	− 1.00	0.03	0.064	− 31.69	− 58.53	− 4.84	**0.021**
2	− 67.89	− 165.11	29.33	0.171	− 0.07	− 0.50	0.37	0.770	− 35.52	− 60.34	− 10.71	**0.005**

Estimates (B) and 95% confidence intervals (CI) from general estimating equations adjusted for residence during pregnancy, gravidity, maternal age, sex of offspring, and maternal pre-pregnancy BMI. Stillborns excluded. p < 0.05 bolded. *ref* reference group, *CI* confidence interval, *IPTp-SP* intermittent preventative treatment in pregnancy with sulfadoxine-pyrimethamine. Intercepts were included in each of the nine multivariable models (not shown)

#### Parasite density

When parasite density was severe as opposed to no parasites observed, birth weight was 410 g lighter (p < 0.001), birth length was 1.4 cm shorter (p < 0.001), and placental weight was 65 g lighter (p < 0.001). Moderate parasite densities were not associated with birth length (p = 0.83) or placental weight (p = 0.082), but were associated with lower birth weight by 228 g (p = 0.016). Mild parasite densities were not statistically distinguishable from no parasites observed for all three birth outcomes (p > 0.2) (Table 4).

#### SP doses

Compared with births to women who received at least three doses of SP, newborns from women who received no doses of SP were 285 g lighter (p = 0.032), 2 cm shorter (p = 0.004), and their placentas were 75 g lighter (p = 0.002). Placentas from women who received one or two doses of SP were 32 g lighter (p = 0.021) and 36 g lighter (p = 0.005), respectively, compared to placentas from women who received three or more doses of SP. Birth weight was lower for women who received one or two doses of SP compared to three or more doses but the difference was not statistically significant (p ≥ 0.17). There was a trend toward shorter birth lengths by half a centimeter if the mother received one instead of at least three doses of SP (B = -0.49, p = 0.064), but no evidence for a difference at two doses instead of three or more (B = -0.07, p = 0.770) (Table 4).

When birth weight was modelled as a dichotomous instead of a continuous variable, chronic infections were associated with a 46% increase in the odds of LBW (OR = 1.46, p < 0.001) and moderate and severe parasite densities were associated with a 35% (OR = 1.35, p = 0.016) and 71% (OR = 1.71, p < 0.001) increase in the odds of LBW, respectively. Receiving only one or two doses of SP compared with three doses was associated with a 14% increase in the odds of LBW (one dose: OR = 1.14, p = 0.023; two doses: OR = 1.14, p = 0.015) (Table 5).

**Table 5:** Associations between PM stage, parasite density, doses of IPTp-SP, and low birth weight (< 2500 g) in three multivariable models

		95% CI		
	OR	Lower	Upper	p-value
Infection stage (ref. None) (N = 305)				
Acute	1.10	0.88	1.37	0.398
Chronic	1.46	1.25	1.70	** < 0.001**
Past	1.10	0.99	1.21	0.068
Parasite density (ref. None) (N = 305)				
Mild	1.05	0.90	1.24	0.534
Moderate	1.35	1.06	1.72	**0.016**
Severe	1.71	1.41	2.06	** < 0.001**
IPTp-SP doses (ref. 3−) (N = 310)				
None	1.22	0.96	1.55	0.100
1	1.14	1.02	1.28	**0.023**
2	1.14	1.03	1.27	**0.015**

Odds Ratios (OR) and 95% confidence intervals (CI) from general estimating equations for logistic regression adjusted for residence during pregnancy, gravidity, maternal age, sex of baby, and maternal pre-pregnancy BMI. p < 0.05 bolded. *ref* reference group, *CI* confidence interval, *IPTp-SP* intermittent preventative treatment in pregnancy with sulfadoxine-pyrimethamine. Intercepts were included (not shown)

### Predictors of SP doses

The characteristics of women who received no doses of SP, representing poor compliance with Malian national policy (Table 6: Model 5) were modelled, as well as the attributes of women who received ≥ 3 doses, indicative of the best compliance (Table 6: Model 6). For each additional year of age, a woman’s odds of receiving no doses of SP decreased by 28% (p = 0.04), and if they had attended primary school instead of receiving no formal education, they were 81% less likely to get no doses of SP (p = 0.03). If they gave birth in 2016 (compared to 2018), they were 79% less likely to get no doses of SP (p = 0.03) (Table 6: Model 5). The only variable that predicted getting ≥ 3 doses of SP was socio-economic status (Table 6: Model 6). Specifically, a one standard deviation increase in the wealth z-score of a woman’s family was associated with 40% higher odds (p = 0.04) of receiving ≥ 3 doses of SP compared to < 3 doses (Table 6: Model 6), but other characteristics of the mother (age, pre-pregnancy BMI, gravidity, residence, education) were not significantly associated with receipt of > 3 doses.

**Table 6:** Multivariable models of maternal and other characteristics associated with the number of SP doses received during pregnancy

	Model 5				Model 6			
		95% CI				95% CI		
	OR	Lower	Upper	p-value	OR	Lower	Upper	p-value
Maternal age (years)	0.72	0.52	0.98	**0.037**	0.95	0.81	1.11	0.520
Pre-pregnancy BMI	1.03	0.82	1.30	0.773	1.06	0.94	1.19	0.385
Gravidity (ref. Multigravid)								
Primigravid	0.61	0.18	2.05	0.422	0.90	0.49	1.65	0.733
Residence during pregnancy (ref. Village)								
Bamako	0.40	0.08	2.06	0.273	1.31	0.64	2.72	0.460
Wealth z-score	0.73	0.42	1.27	0.267	1.40	1.02	1.92	**0.036**
Education (ref. Never attended)								
Primary	0.19	0.04	0.83	**0.027**	1.42	0.69	2.91	0.339
Secondary	0.58	0.17	1.97	0.385	1.42	0.66	3.06	0.369
Beyond secondary	2.45	0.64	9.46	0.193	1.25	0.49	3.19	0.640
Season of birth (ref. Hot Dry, Mar–May)								
Rainy, Jun–Oct	2.45	0.45	13.36	0.300	0.93	0.46	1.90	0.839
Cool Dry, Nov–Feb	5.28	1.00	28.02	0.051	0.72	0.35	1.47	0.364
Year of birth (ref. 2018)								
2014	0.16	0.02	1.26	0.082	0.80	0.30	2.13	0.652
2015	0.31	0.06	1.73	0.184	0.72	0.27	1.89	0.502
2016	0.21	0.05	0.84	**0.028**	0.70	0.30	1.63	0.413
2017	0.24	0.05	1.09	0.064	0.73	0.32	1.68	0.464
2019	1.22	0.28	5.23	0.794	1.87	0.69	5.09	0.221

Model 5 (poor SP uptake): No doses received (coded as 1) versus 1 + doses (coded as 0). Model 6 (best SP uptake): 3 + doses (coded as 1) versus < 3 doses (coded as 0) (N = 317)

Odds ratios (OR) and 95% confidence intervals (CI) from general estimating equations for logistic regression. p < 0.05 bolded. *ref* reference group, *CI* confidence interval. Intercepts were included (not shown)

## Discussion

### Rural versus urban residence

Placental malaria (PM) was evaluated in 317 singleton births to 249 mothers who participated in a longitudinal cohort in a rural community on the Bandiagara Escarpment in Central Mali. Eighty-four percent of the births were to women who continued to live in the rural community where they were followed from enrollment (1998 to 2000) to the time they gave birth (2011–2019). Sixteen percent of the births were to women in the cohort who had migrated to Bamako. The odds of a placenta being infected with malaria (including both active and past infections) were 67% lower (p = 0.015) for Bamako compared to the villages–after adjusting for other covariates such as the number of SP doses the woman received during pregnancy. Similarly, the prevalence of *P. falciparum* in children aged 6 to 59 months was far higher in the Mopti Region than in Bamako in the Demographic and Health Surveys in the years 2010–2018 [9]. Prevalence of malaria is generally higher in rural compared to urban areas [15, 16], although a few studies reported no difference between rural and urban locations [17, 18]. Differences in infrastructure, or social and environmental factors that might contribute to the lower prevalence of placental malaria in Bamako were not investigated. However, one advantage of the current study is that the women at both locations came from the same ethnicity (Dogon), reducing genetic or cultural differences that may influence malaria susceptibility. Moreover, as they came from the same cohort, they were similar in age and shared similar childhood and adolescent environments. Those who migrated to Bamako did so at a mean (SD) age of 17.9 (3.1) years.

### PM prevalence

The overall prevalence of PM was 71%, similar to other regions in Sub-Saharan Africa where malaria is endemic (for example, 75% in southeastern Tanzania [19], 59% in Sudan [20]). The prevalence of past infections, at 48%, was higher than for acute (5%), chronic (18%), and no (29%) infection. Since far more past infections were identified than active ones (acute and chronic), this study had more statistical power to detect risk factors for active and past infections combined than for active infections alone. Malaria parasites were not detected in most placentas (77%), and when they were detected, the infections were mostly mild as opposed to moderate or severe, similar to a study in Kenya [21] that also used scoring criteria based on Bulmer et al. [13] and Muehlenbachs et al. [12]. The authors are not aware of any other studies conducted in Mali that examined PM prevalence using placental tissue histology, although several have used placental blood smears [22–24].

### Year of offspring birth

Several variables were strongly associated with PM. In particular, the odds of placental malaria infection were much higher for births to women in the earlier years of the study compared with the later years, which may have reflected increased anti-malaria efforts over time. For example in 2015, the odds for malaria infection (active and past combined) were eight-fold higher (p = 0.001) than in 2018. In 2015, the Mopti Region had twice the malaria prevalence, compared to the national average, for children under five years, prompting an indoor residual spraying program that took place in 2017 and included the Bandiagara Escarpment [25]. From 2016 to 2017, peak malaria incidence decreased by 42%, on average, in sprayed health facility catchment areas compared to non-sprayed communities in the Mopti Region [25]. Spraying campaigns also took place in 2018 [26] and 2019 [27]. The current study provides additional evidence for the success of malaria control campaigns on the Bandiagara Escarpment [28].

### Confounding between maternal age and year of offspring birth

Younger maternal age (< 20 years) has been reported to be associated with PM in Mali [24] and a study in the District of Bandiagara in 1993 and 1994 reported that women under the age of 27 years had more malaria parasites in blood smears [29]. However, after adjusting for year of study in an attempt to control for changes in yearly exposure to malaria, maternal age was no longer associated with PM in the current study (Table 2 and 3). Although maternal age and the year in which a woman gave birth were correlated, birth year was the stronger predictor of PM. Other studies tend not to adjust for year of birth, which is not problematic if either risk of infection is known to be constant across years, or if the study is cross-sectional. As neither of these conditions applied here, it was important to adjust for offspring’s birth year as was done in Model 1. Participants were young (age range 15.5–25.8 with a median of 20.4 years, N = 249) (Table 1), whereas other studies that detect a stronger association between PM and maternal age may have had a wider age range of women.

### Gravidity

Previous studies reported greater PM risk among women who were primigravida [20, 30–32]. This finding was replicated for active and past PM infections only if maternal age and year of birth, which are associated with gravidity (Additional file 2: Fig. S2), were not included in the model (Additional file 4: Table S1). It has been reported that the timing of malaria infection during pregnancy differentially impacts primigravid and multigravid women [33], but this possibility cannot be assessed in this study as data on the timing of infection were not collected.

### Maternal BMI

A one unit increase in maternal pre-pregnancy BMI was associated with a 13% decrease in the odds of active and past PM (Table 2: Model 1). The mean BMI for the multigravida was 0.7 kg/m^2^ greater than for the primigravida (Additional file 2: Fig. S2). However, the inverse association detected between BMI and PM is unlikely to be due to confounding with gravidity because the models adjusted for gravidity. In Tanzania, underweight women had decreased risk of sub-microscopic placental malaria infection, while overweight or obese women had higher odds of placental malaria by blood smear compared to normal weight women [34]. The authors speculated that iron deficiency in the underweight mothers may have protected them against PM. In the current study, anaemia was common, but most of the placentas were from women who had normal BMI (84.5% normal (N = 268), 4.1% underweight (N = 13), 11.4% overweight/obese (N = 36). One possibility is that women of higher BMI were more sedentary and engaged in less outdoor manual labour and thus had less exposure to mosquitoes.

### Maternal education

Mothers who had some education beyond secondary school had decreased risk of both active and past PM infections combined. Similarly, in other studies education has been reported to be associated with decreased risk of malaria in pregnancy [35, 36]. However, associations between education and PM were not found in Uganda [35] and Sudan [20]. Education has been associated with health-seeking behaviours, such as prenatal visits and optimal SP dosing [37]. As the models in the current study adjust for SP dosing, it is also possible that the better educated mothers performed less manual outdoor labour and had less exposure to mosquitoes.

### Season of birth

The odds of PM (active and past infections combined) were 77% lower for births in the rainy season (June through October) compared to the hot dry season (March through May) (Table 2: Model 1). Similarly, in The Gambia and Burkina Faso, risk for active and past PM was lower for births in the rainy season compared to the dry season [38]. The odds of active PM infections (excluding past infections) were 11-fold greater for births during the rainy and seven-fold greater for births during the cool dry season compared to births in the hot dry season (Table 3: Model 3). This result may reflect increased transmission of malaria during the rainy season, instigating active infections. Similarly, using thick blood smears, increased PM was found in the rainy season in Koro and Bandiagara in Mali [24].

### Uptake of SP doses

The 2018–2022 National Strategy for Malaria Control in Mali calls for at least 80% of pregnant women to receive ≥ 3 doses of sulfadoxine-pyrimethamine (SP) during their pregnancies [39]. At least 3 SP doses have been recommended to support and protect the period of rapid fetal weight gain during the third trimester [24] and have been associated with full term births and normal birth weights [40]. However, only 25% of women received ≥ 3 doses of SP, 36% received two doses, 32% received one dose, and 7% received no doses of SP. Thus dosing of SP fell far short of national guidelines.

Across several regions of Mali in 2015, 66% of women reported taking SP during pregnancy. Of those who took SP, 63% reported < 2 and 37% reported taking ≥ 3 doses [41]. Compared with that study, in this cohort there was a lower prevalence of women who received no doses or who received ≥ 3 doses of SP. Moreover, the first SP dose occurred late in pregnancy at an estimated mean (SD) gestational age of about 26 (10) weeks (see Methods), which is at the end (week 26) rather than the beginning (week 13) of the second trimester when dosing can begin. Late first SP doses (after 21 weeks) may provide suboptimal protection against infection as was seen in Benin [42]. In rural northern Ghana, women who had a second dose of SP during the 2nd trimester were more likely to receive ≥ 3 doses compared to women whose first dose was delayed to the third trimester [40]. A difference in dosing in Bamako compared to the villages was not detected, and it would be desirable for dosing at both sites to start earlier in the second trimester of pregnancy.

### PM and SP doses

Compared with mothers who received ≥ 3 doses of SP, placentas from mothers who received no doses had 14-fold higher (p = 0.001) odds of active and past PM (Table 2: Model 1). This was a stark difference. The odds for active (excluding past) infections were ten-fold higher (p = 0.001) for women who had no doses and more than two-fold higher for women who had one dose (p = 0.036) compared to ≥ 3 doses (Table 3: Model 3). However, regardless of whether active and past infections were combined, or whether only active infections were considered, no difference was detected in the odds of PM between receipt of 2 versus ≥ 3 SP doses (Table 2 and 3). This finding contrasts with an earlier study in 2006–2008 in the Segou Region of Mali which demonstrated two-fold lower prevalence of PM by placental blood smear with 3 SP doses compared to 2 doses after adjustment for gravidity, season of birth, maternal age, and malaria at enrollment [23]. A meta-analysis of 6 sub-Saharan countries found a 49% reduced risk of PM with ≥ 3 compared to 2 SP doses [43], but this finding was restricted to primigravid and secundigravid women and did not pertain to women’s subsequent pregnancies. In a Tanzanian low malaria transmission setting, no difference in PM was found for women who had ≥ 3 doses compared to 2 doses [44]. However, the Tanzanian study found that risk for maternal anaemia was 36% higher in women who received 2 instead of ≥ 3 SP doses, highlighting the drug’s role in combating malaria in the peripheral blood.

### Birth outcomes and SP doses

Birth outcomes were examined in relation to the number of SP doses a pregnant woman received (Table 4). Compared with women who received ≥ 3 doses, birth weight was lower by 285 g (p = 0.03) in women who received no doses of SP. It was lower by 75 and 68 g in women who received one or two doses, respectively, but these latter two differences were not statistically significant (p > 0.17) compared with ≥ 3 doses. When birth weight was dichotomized as low versus normal, the risk of low birth weight (LBW) was 14% higher for one and two SP doses compared to ≥ 3 doses (p = 0.02) and 22% higher for no doses versus ≥ 3 but the p-value was 0.1 for this last comparison (Table 5).

In South West Cameroon, ≥ 3 doses were associated with lower odds of LBW compared to ≤ 1 dose, but unlike the current study, no difference was detected between ≥ 3 doses and 2 doses [45]. In Southeast Tanzania, higher birth weight was seen with ≥ 3 doses compared to two doses, as well as lower risk of LBW [46]. Lower risk of LBW was also seen in Nigeria with 3 doses compared to 2 doses [47]. A meta-analysis showed a stronger association between mean birth weight and 3 doses than mean birth weight and 2 doses [43]. In the current study, higher birth weights were not detected when women received ≥ 3 compared to 2 doses, but increased risk of LBW was observed with 2 compared to ≥ 3 doses. A topical review of LBW in Eastern Africa [48] found that ≥ 3 doses of SP was associated with decreased risk of LBW and increased birth weight compared to 2 doses. This review also addressed evidence that SP may not protect against risk of LBW in areas of high SP resistance. Parasite molecular markers demonstrating SP resistance were associated with decreased effectiveness of SP in preventing malaria infections and LBW in a meta-analysis of 57 studies in sub-Saharan Africa [49]. SP resistance in Mali increased 7% from 2000 to 2020 [50], a rate lower than for some East African countries such as Mozambique (64%) and Tanzania (55%) but higher than for other West African countries such as Nigeria (-14%) and Burkina Faso (0.13%).

In the current study, birth length was 2 cm shorter (p = 0.004) when no SP doses were received compared to ≥ 3 doses. A trend toward shorter birth length by half a centimeter for one instead of ≥ 3 SP doses (B = − 0.49, p = 0.064) was observed, but there was no evidence for a difference at two doses instead of ≥ 3 doses (B = − 0.07, p = 0.770). In Ghana doses even in excess of 5 were not associated with a dichotomous variable for birth length [40]. In Malawi, the child’s length at 4 weeks of age was greater if the mother received SP doses on a monthly as opposed to an intermittent basis [51].

### Placental weight

The mean (SD) placental weight in the current study was 483 (93) g. Placentas were 75 g lighter (p = 0.002) from women who received no SP doses compared to ≥ 3 doses. Placentas from women who received one or two doses were 32 g lighter (p = 0.021) and 36 g lighter (p = 0.005), respectively, compared to placentas from women who received ≥ 3 doses. Thus, placental weight was higher at any level of dosing, compared to no dosing, and one or two doses could be distinguished from three doses but not one dose from two doses. In southern Mozambique, placental weight was 49 g heavier and the duration of pregnancy was 6.1 days longer in women who had two SP doses compared to no doses [52]. Elsewhere, the impact of SP dosing on placental weight is largely unreported, although malaria infections in peripheral blood early in pregnancy were associated with decreased placental weight compared to uninfected controls in Tanzania [53].

### SP uptake

Some women received no doses of SP (Table 6: Model 5), which put their offspring at high risk for poor birth outcomes. These women tended to be younger and they were more likely to have had no formal education instead of having gone to primary school. These results underscore the importance of primary school education for girls. This finding was similar to results from survey data for twelve sub-Saharan African countries including Mali in the years 2015–2019, showing that maternal education and maternal age were positively associated with SP doses [54]. The current study also identified a trend toward 5 times increased odds of receiving no SP doses (p = 0.06) if the birth was in the cool dry season instead of the hot dry season. Further research would be needed to understand whether this seasonal difference is real and, if applicable, any underlying causes. One possibility is that this finding might reflect seasonal differences in women’s workload impinging on their ability to seek antenatal care. Women who gave birth in 2018 and 2019 as opposed to earlier years were more likely to get no doses of SP, which is surprising and bears further investigation (Table 6: Model 5).

In the current study, socio-economic status was the only variable that predicted getting ≥ 3 doses of SP. For each additional increase in the wealth z-score of a woman’s family, the woman was 40% more likely to receive ≥ 3 doses of SP (Table 6: Model 6). Moreover, younger women were more likely to receive no doses of SP, but older women were not found to be more likely to receive > 3 doses. A study conducted on the Bandiagara Escarpment in Mali in 2015 [41] reported that women under age 20 years were less likely to receive ≥ 3 doses of SP. The Bandiagara study [41] was consistent with the current study in not finding an association between SP uptake and urban/rural residence, but it differed in that it did not find an association between maternal education or socioeconomic status and SP doses. Similar to the findings of the current study, greater affluence was associated with uptake of ≥ 3 doses of SP in Uganda [55] and Nigeria [56].

### Study limitations

An important limitation of this study is its observational, non-randomized design. Therefore, to improve the comparison of PM in Bamako versus the villages, it was helpful that the participants in both places came from the same longitudinal cohort and the same ethnicity. Unlike most studies that entail an urban—rural comparison, this study was restricted to women who belonged to a specific cohort established by BIS in 1998 to 2000 in a rural community on the Bandiagara Escarpment. No placentas came from women who were not part of this cohort. Although the cohort study as a whole had unusually strong retention of participants who migrated to Bamako, losing only 6% of urban migrants to follow-up, participation in the placental collections in Bamako was lower than in the villages. Placental collection in Bamako was logistically challenging as the women in the cohort gave birth at a variety of hospitals and clinics, whereas in the rural community only one hospital was involved.

Another limitation is that data on gestational age at delivery were not available. Moreover, maternal anaemia was not evaluated, which is known to be associated with malaria in pregnancy, and peripheral blood parasitaemia levels were not measured. Lastly, PM was assessed through the histological examination of the intervillous space of placental samples, which is an established method for assessment of PM. However, it is likely to miss early or low density (sub-microscopic) infections that would require molecular analysis for detection. Data were also lacking on the onset of malarial infection, which would have been useful for shedding light on associations between placental malaria and season of birth.

## Conclusions

The odds of a placenta being infected with malaria were 67% lower in Bamako compared to a set of rural Dogon villages on the Bandiagara Escarpment in Mali. The women at both locations came from the same prospective cohort study, reducing confounding by genetic or cultural differences in vulnerability to malaria. Consistent with increased malaria control efforts on the Bandiagara Escarpment, PM infection decreased substantially from 2014 to 2018. Women whose pre-pregnancy BMI was higher and who had some education beyond secondary school had decreased risk for PM. Only 25% of women received the recommended 3 or more doses of SP, and these women tended to come from wealthier families but were not more likely to live in Bamako. Thus in both locations, dosing of SP fell far short of national guidelines. A great improvement in birth outcomes (+ 285 g birth weight, + 2 cm birth length, + 75 g placental weight) was found for women who had 3 doses of SP compared to no doses, but a difference in birth weight or length for women who had 3 instead of 2 doses of SP was not detected. However at 2 instead of ≥ 3 doses placentas were 36 g lighter and the odds of LBW as a binary variable were 14% higher. This study provides insight into how to target women at risk for receiving no SP during pregnancy: they tend to be younger and to lack primary school education. Seeking out women who have these characteristics and promoting their access to antenatal care as early as possible in the second trimester will likely have a positive impact on birth outcomes. It is also desirable for women to get ≥ 3 doses, but high priority should be placed on improving access to antenatal care for women who otherwise will get no doses of SP. In this study, such women comprised 7% of the sample and their neonates were 285 g lighter and 2 cm shorter.

## Supplementary Information

**Additional file 1: Fig. S1.** Relationship between maternal age and date of offspring birth. Linear fit line is bold; thinner lines indicate 95% confidence intervals around the mean.

**Additional file 2: Fig. S2.** Box plots of maternal age (**A**), offspring year of birth (**B**), and maternal pre-pregnancy BMI (**C**), by gravidity (multigravida or primigravida).

**Additional file 3: Fig. S3.** Numbers and proportion of PM infection stages by offspring month of birth for all study years. Seasons are indicated by colored bars as cool/dry (blue), hot/dry (red) and rainy (green).

**Additional file 4: Table S4.** Multivariable model of maternal risk factors for PM infection (active and past infections combined) with maternal age omitted (N = 313)

## Data Availability

The dataset generated and analyzed during this study will be made available at ICPSR (Project ID: DSDR-163381) at the University of Michigan.

## Abbreviations

ANC: Antenatal care
IPTp-SP: Intermittent preventive treatment in pregnancy with sulfadoxine-pyrimethamine
PM: Placental malaria
SP: Sulfadoxine-pyrimethamine
