# Supplementary figures and images for: Risk factors for placental malaria, sulfadoxine-pyrimethamine doses, and birth outcomes in a rural to urban prospective cohort study on the Bandiagara Escarpment and Bamako, Mali
Source: Malar J. 2022 Mar 31;21:110. doi: 10.1186/s12936-022-04125-6 (PMC8974163; doi:10.1186/s12936-022-04125-6)

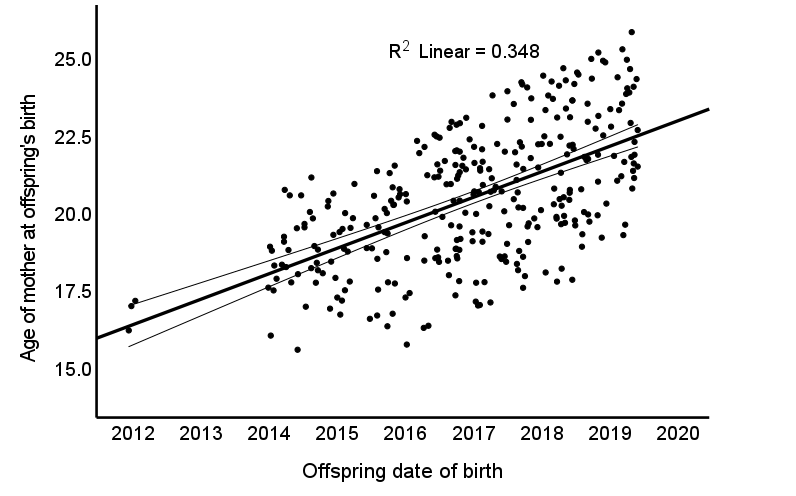


R2 = 0.35

Supplement: Supplementary file 1 — Additional file 1: Fig. S1. Relationship between maternal age and date of offspring birth. Linear fit line is bold; thinner lines indicate 95% confidence intervals around the mean. [file 12936_2022_4125_MOESM1_ESM.docx]

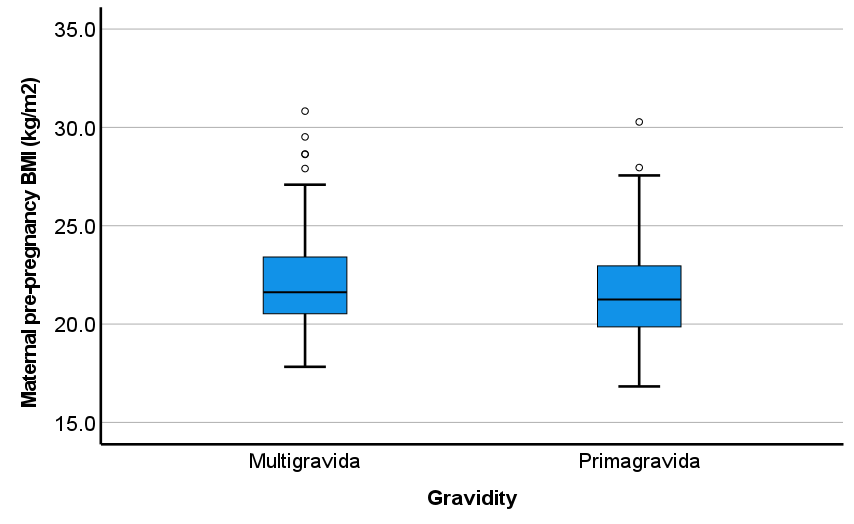

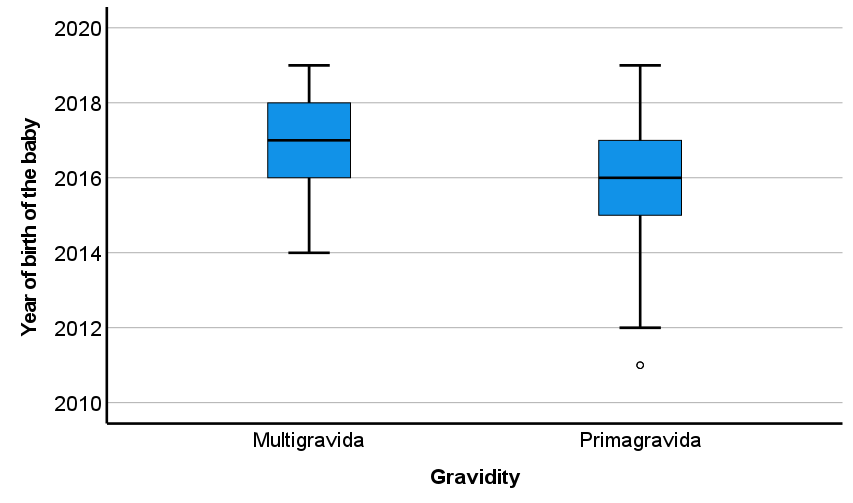

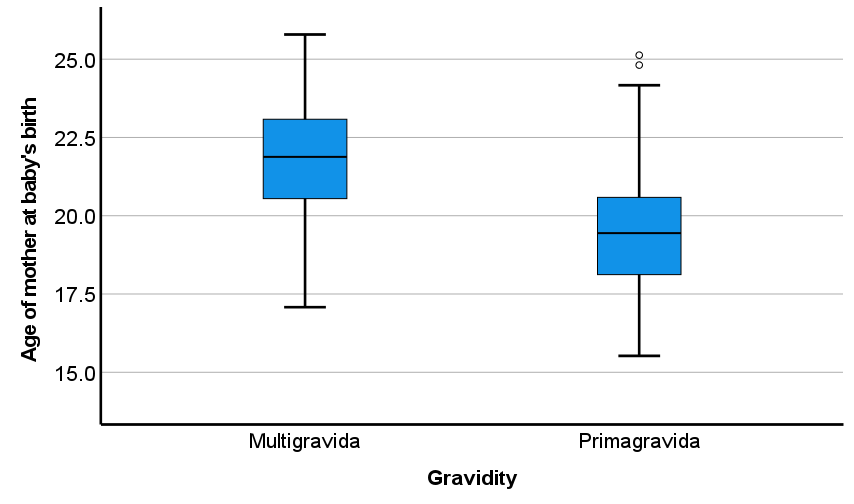


**A**

**B**

**Offspring year of birth**

**Age of mother at offspring’s birth**

**C**

**Maternal pre-pregnancy BMI (kg/m2)**

Supplement: Supplementary file 2 — Additional file 2: Fig. S2. Box plots of maternal age (A), offspring year of birth (B), and maternal pre-pregnancy BMI (C), by gravidity (multigravida or primigravida). [file 12936_2022_4125_MOESM2_ESM.docx]
