# Supplementary material for: Risk factors for placental malaria, sulfadoxine-pyrimethamine doses, and birth outcomes in a rural to urban prospective cohort study on the Bandiagara Escarpment and Bamako, Mali
Source: Malar J. 2022 Mar 31;21:110. doi: 10.1186/s12936-022-04125-6 (PMC8974163; doi:10.1186/s12936-022-04125-6)
Supplement: Supplementary file 3 — Additional file 3: Fig. S3. Numbers and proportion of PM infection stages by offspring month of birth for all study years. Seasons are indicated by colored bars as cool/dry (blue), hot/dry (red) and rainy (green). [file 12936_2022_4125_MOESM3_ESM.docx]

| **Month** | **N** | | | | | **Proportion/month** | | | | |
| --- | --- | --- | --- | --- | --- | --- | --- | --- | --- | --- |
|  | None | Acute | Chronic | Past | Total | None | Acute | Chronic | Past | Total |
| Jan | 6 | 1 | 3 | 24 | 34 | 0.176 | 0.029 | 0.088 | 0.706 | 1.000 |
| Feb | 6 | 0 | 6 | 8 | 20 | 0.300 | 0.000 | 0.300 | 0.400 | 1.000 |
| Mar | 8 | 0 | 1 | 17 | 26 | 0.308 | 0.000 | 0.038 | 0.654 | 1.000 |
| Apr | 7 | 1 | 1 | 23 | 32 | 0.219 | 0.031 | 0.031 | 0.719 | 1.000 |
| May | 8 | 0 | 0 | 12 | 20 | 0.400 | 0.000 | 0.000 | 0.600 | 1.000 |
| Jun | 13 | 3 | 2 | 12 | 30 | 0.433 | 0.100 | 0.067 | 0.400 | 1.000 |
| Jul | 7 | 1 | 1 | 8 | 17 | 0.412 | 0.059 | 0.059 | 0.471 | 1.000 |
| Aug | 8 | 3 | 5 | 13 | 29 | 0.276 | 0.103 | 0.172 | 0.448 | 1.000 |
| Sept | 11 | 3 | 13 | 8 | 35 | 0.314 | 0.086 | 0.371 | 0.229 | 1.000 |
| Oct | 6 | 3 | 12 | 9 | 30 | 0.200 | 0.100 | 0.400 | 0.300 | 1.000 |
| Nov | 5 | 1 | 7 | 8 | 21 | 0.238 | 0.048 | 0.333 | 0.381 | 1.000 |
| Dec | 5 | 1 | 4 | 7 | 17 | 0.294 | 0.059 | 0.235 | 0.412 | 1.000 |
| Total | 90 | 17 | 55 | 149 | 311 |  | | | | |
