# Supplementary material for: Risk factors for placental malaria, sulfadoxine-pyrimethamine doses, and birth outcomes in a rural to urban prospective cohort study on the Bandiagara Escarpment and Bamako, Mali
Source: Malar J. 2022 Mar 31;21:110. doi: 10.1186/s12936-022-04125-6 (PMC8974163; doi:10.1186/s12936-022-04125-6)
Supplement: Supplementary file 4 — Additional file 4: Table S4. Multivariable model of maternal risk factors for PM infection (active and past infections combined) with maternal age omitted (N = 313) [file 12936_2022_4125_MOESM4_ESM.docx]

**SI Table 1** Multivariable model of maternal risk factors for PM infection (active and past infections combined) with maternal age omitted (N =313

|  | OR | 95% CI | | p-value |
| --- | --- | --- | --- | --- |
|  |  | Lower | Upper |  |
| Pre-pregnancy BMI | 0.86 | 0.76 | 0.97 | **0.018** |
| Gravidity (ref. Multigravid) |  |  |  |  |
| Primigravid | 1.88 | 1.09 | 3.26 | **0.023** |
| IPTp-SP Doses (ref. 3+) |  |  |  |  |
| None | 7.30 | 1.54 | 34.50 | **0.012** |
| 1 | 2.26 | 1.15 | 4.45 | **0.018** |
| 2 | 1.58 | 0.79 | 3.16 | 0.198 |
| Residence during pregnancy (ref. Village) |  |  |  |  |
| Bamako | 0.30 | 0.15 | 0.63 | **0.001** |
| Wealth z-score | 1.31 | 0.96 | 1.79 | 0.089 |
| Education (ref. Never attended) |  |  |  |  |
| Primary | 0.55 | 0.27 | 1.12 | 0.097 |
| Secondary | 0.98 | 0.43 | 2.19 | 0.954 |
| Beyond secondary | 0.21 | 0.08 | 0.53 | **0.001** |
| Season of offspring birth (ref. Hot Dry, Mar – May) |  |  |  |  |
| Rainy, Jun – Oct | 0.76 | 0.39 | 1.48 | 0.420 |
| Cool Dry, Nov – Feb | 1.08 | 0.50 | 2.36 | 0.842 |

Estimates (B) and Odds Ratios (OR) from general estimating equations (95% confidence interval) for logistic regressions, p < 0.05 bolded. *ref* reference group, *CI* confidence interval, *IPTp-SP* intermittent preventative treatment in pregnancy with sulfadoxine-pyrimethamine. Intercept was included (not shown).
